# Risk of Accidents or Chronic Disorders From Improper Use of Mobile Phones: A Systematic Review and Meta-analysis

**DOI:** 10.2196/21313

**Published:** 2022-01-20

**Authors:** Xinxi Cao, Yangyang Cheng, Chenjie Xu, Yabing Hou, Hongxi Yang, Shu Li, Ying Gao, Peng Jia, Yaogang Wang

**Affiliations:** 1 School of Public Health Tianjin Medical University Tianjin China; 2 School of Public Administration Nanjing Normal University Nanjing China; 3 Tianjin Medical University General Hospital Tianjin Medical University Tianjin China; 4 School of Resource and Environmental Sciences Wuhan University Wuhan China; 5 International Institute of Spatial Lifecourse Epidemiology (ISLE) Wuhan University Wuhan China

**Keywords:** cell phone, mobile phone, accident, neoplasm, radiation

## Abstract

**Background:**

Mobile phone use has brought convenience, but the long or improper use of mobile phones can cause harm to the human body.

**Objective:**

We aimed to assess the impact of improper mobile phone use on the risks of accidents and chronic disorders.

**Methods:**

We systematically searched in PubMed, EMBASE, Cochrane, and Web of Science databases for studies published prior to April 5, 2019; relevant reviews were also searched to identify additional studies. A random-effects model was used to calculate the overall pooled estimates.

**Results:**

Mobile phone users had a higher risk of accidents (relative risk [RR] 1.37, 95% CI 1.22 to 1.55). Long-term use of mobile phones increased accident risk relative to nonuse or short-term use (RR 2.10, 95% CI 1.63 to 2.70). Compared with nonuse, mobile phone use resulted in a higher risk for neoplasms (RR 1.07, 95% CI 1.01 to 1.14), eye diseases (RR 2.03, 95% CI 1.27 to 3.23), mental health disorders (RR 1.16, 95% CI 1.02 to 1.32), and headaches (RR 1.25, 95% CI 1.18 to 1.32); the pooled risk of other chronic disorders was 1.20 (95% CI 0.90 to 1.59). Subgroup analyses also confirmed the increased risk of accidents and chronic disorders.

**Conclusions:**

Improper use of mobile phones can harm the human body. While enjoying the convenience brought by mobile phones, people have to use mobile phones properly and reasonably.

## Introduction

In the first quarter of 2019, the number of mobile phone users reached 7.9 billion, with an increase of approximately 2% year-on-year [1]. China had the most net additions during this quarter (30 million), followed by Nigeria (5 million), and the Philippines (4 million). In addition, it was predicted that the worldwide mobile phone market would reach 1.5 billion shipment units by the end of 2019 and that the pending arrival of 5G would attract more phone users by 2020 or 2021 [2]. Although mobile phones facilitate people's daily lives and provide effective auxiliary means for the treatment and management of diseases [3-5], the health hazards potentially caused by using mobile phones are also a growing concern.

Although many countries and regions have passed laws prohibiting the use of mobile phones while driving, the number of reported traffic accidents caused by using mobile phones while driving has been increasing in recent years [6,7]. Nearly one-quarter of all traffic accidents in the United Kingdom in 2013 were caused by drivers using phones while driving. In addition, harm may be caused to the ears, parotid glands, and indirect brain areas during mobile phone usage [8-10]. Some in vivo or in vitro [11], simulator [12], or real-world studies [13] have been carried out to test the effects between human body and mobile phone use. There is currently no consensus on the use of mobile phones and chronic disorders, especially with respect to neoplasms, because results conflict. Mobile phone radiation has been classified as a possible carcinogen to humans [14]; radiation might cause tumors or accelerate the growth of subclinical tumors [15,16]. In recent years, head and neck injuries related to mobile phones have increased sharply [17]. A cross-sectional study [17] in the United States using a national database showed that mobile phone use can be distracting and cause injuries. In addition, increasing attention had been paid to the impact of mobile phone use on mental health (for example, addiction [18,19]), and a new term—*nomophobia*—which is short for *no mobile phone phobia* and has been considered as a symptom or syndrome of problematic digital media use in mental health [20]. However, some studies believe that the available evidence has not yet suggested that mobile phone use can cause damage to the human body (especially with respect to cancer).

Given that the use of mobile phones is growing rapidly, it is still doubted whether the improper use of mobile phones causes injuries to the human body. Our paper will provide a thorough review of literature to explore the impact of improper mobile phone use, which includes accidents and chronic disorders, on human body health.

## Methods

### Search Strategy

Two of the authors systematically searched PubMed, EMBASE, Cochrane, and Web of Science databases from inception to April 4, 2019. The search was limited to studies on the human body published in the English language. Additional literature was screened by manually searching the reference lists of recent reviews and studies for papers meeting the inclusion criteria.

### Inclusion and Exclusion Criteria

According to the International Statistical Classification of Diseases, tenth revision [21], accidents are defined as unplanned events that sometimes have inconvenient or undesirable consequences, at times being inconsequential, and which include transport accidents and other injuries. In our study, we used the term *chronic disorders* for all nonaccident outcomes, including neoplasms (brain tumor, thyroid cancer, glioma and astrocytoma); mental health disorders such as attention-deficit/hyperactivity disorder (ADHD), nomophobia-anxiety, insecurity, anger, or discomfort; headaches; sleep disorders; injuries to the head (eye, ear, oral); injuries to the wrist; diseases of male genital organs; and other unspecific disorders including DNA damage, genotoxic effects, blood–cerebrospinal fluid barrier damage, serum S100B levels damage, total prostate specific antigen (tPSA) disorder, free prostate specific antigen (fPSA) disorder, fPSA/tPSA disorder, poor DNA integrity, chromosomal damage. The term *nomophobia*, constructed on the definitions described in the Diagnostic and Statistical Manual of Mental Disorders (Fourth edition) [22] and labeled as a “phobia for a particular/specific thing,” was used to describe the psychological condition when people had a fear of being detached from mobile phone connectivity.

Our inclusion criteria were studies that focused on (1) damage, including accidents and chronic disorders, instead of promoting healthy outcomes; (2) the use of mobile phones, including digital phone and mobile phone radio frequency radiation; (3) improper use of mobile phone, including inappropriate use occasions (eg, using mobile phone while driving or cycling), long-time or long-term use of mobile phone, and using the phone in an incorrect posture; (4) accidents occurring during mobile phone use or chronic disorders resulting from mobile phone use rather than those from any other cause (eg, occupational injuries); and studies that were (5) published in English and with (6) outcome indicators, including odd ratios (OR) or relative risk (RR) and 95% confidence intervals or mean and standard deviation.

Abstracts, comments, conferences, replies, responses, reviews (including systematic reviews), case reports, and animal studies were excluded. Additionally, studies with incomplete data and duplicate studies were also excluded.

### Data Extraction and Quality Assessment

The 2 authors worked simultaneously, but independently, to screen studies, extract data from studies meeting the inclusion criteria, and assess the quality of these studies. Each author’s results were cross-checked by the other, and any disagreements on study selection, data extraction, and study quality assessment were resolved by another author.

The following information was collected using standardized data extraction forms: author information, publication year, study design, participant age, sample size, study area, measures of mobile phone use, measures of outcome-related behavior, and key outcomes.

The Newcastle-Ottawa Scale [23] was designed for the evaluation of case-control studies and cohort studies. The evaluation criteria for cross-sectional studies included 11 items recommended by the Agency for Healthcare Research and Quality [24]. The quality of each study was graded as good, fair, or poor. To be rated as good, studies needed to meet all criteria. A study was rated as poor when 1 (or more) domain was assessed as having a serious flaw. Studies that met some but not all criteria were rated as fair.

### Data Analysis

A random-effects model was used to calculate overall pooled estimates. Tests for heterogeneity between studies’ results were performed with the Cochran Q statistic and were quantified with the *I^2^* statistic.

To examine the robustness of the findings, we performed subgroup analyses by country, participant age, sample size, and study-specific outcomes (accidents and chronic disorders). To validate the robustness of the findings, we performed a sensitivity analysis. The potential for publication bias was graphically explored with funnel plots, and publication bias was tested for significance with the Egger test and Begg test. All statistical procedures were 2-tailed with a significance level of 0.05 and were conducted using Stata software (version 13.0; StataCorp LLC).

## Results

### Study Inclusion

**Figure 1:**
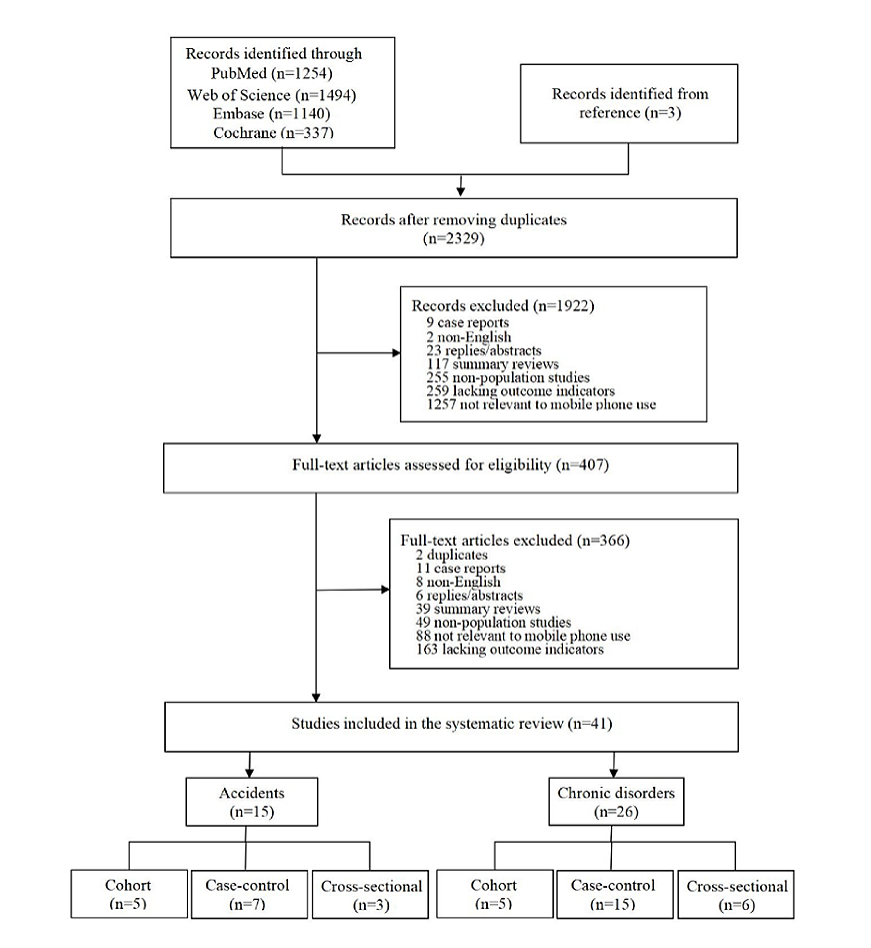
Flowchart of the selection of studies.

A total of 4228 studies were identified by the initial database search, and 3 studies were obtained by searching references; 2329 studies remained after the removal of duplicates (Figure 1). After screening titles and abstracts, 1922 records were excluded because they did not meet the selection criteria: case reports (n=9), summary reviews (n=117), nonpopulation studies (n=255), not about mobile phone use (n=1257), non-English (n=2), replies/abstracts (n=23), and no outcome indicators (n=259). Full texts of remaining papers were assessed for eligibility; 142 records were excluded because they were duplicates (n=2), case reports (n=11), summary reviews (n=39), nonpopulation research (n=49), not about mobile phone use (n=88), not English (n=8), replies/abstracts (n=6), or lacked outcome indicators (n=163). Finally, 41 studies [25-65] were included, which included cohort studies (n=10), case-control studies (n=20), and cross-sectional studies (n=11). Details are presented in Table S1 in Multimedia Appendix 1.

### Study Characteristics and Quality Assessment

Of the 41 papers, 29 papers were published between 2011 and 2019 [25-28,30,31,35-37,41-43,45-48,50,54-65], 11 papers were published between 2002 and 2009 [32-34,38-40,44,49,51-53], and 1 paper was published in 1997 [29]. The sample sizes of the studies ranged from 6 to 15,406,515. All participants were over 7 years old. Studies were carried out in the United States (n=8) [26,30,32,37,45,50,59,64], Sweden (n=5) [38,51-53,61], Canada (n=3) [34,49,56], Korea (n=3) [27,62,63], China (n=2) [25,42], Vietnam (n=2) [31,58], Iran (n=2) [47,54], Denmark (n=1) [28], Italy (n=1) [33], Malaysia (n=1) [46], and Brazil (n=1) [57]; the remaining studies lacked relevant regional information. The outcomes were divided into accidents and chronic disorders—15 studies focused on accidents [30-34,45-47,49,50,54,56-58,64], which were mainly related to transport accidents (car accident, motorcycle accident, and unspecified transport accidents) and other accidental injuries, such as electrical injuries and explosions, and 26 studies [25-29,35-44,48,51-53,55,59-63,65] focused on chronic disorders, including neoplasms, ADHD, nomophobia, headaches, sleep disorders, dry eye diseases, ear injuries, oral problems, wrist injuries, reproductive health issues, and other unspecific chronic disorders (including DNA damage, genotoxic effects, blood-cerebrospinal fluid barrier, serum S100B levels, tPSA, fPSA, fPSA/tPSA, DNA integrity, chromosomal damage). Additional details can be found in Table S2 in Multimedia Appendix 1.

The results of the quality assessment indicated that 16 studies were good quality, and 25 were fair (Table S3 in Multimedia Appendix 1).

### Mobile Phone Use and Accidents

**Figure 2:**
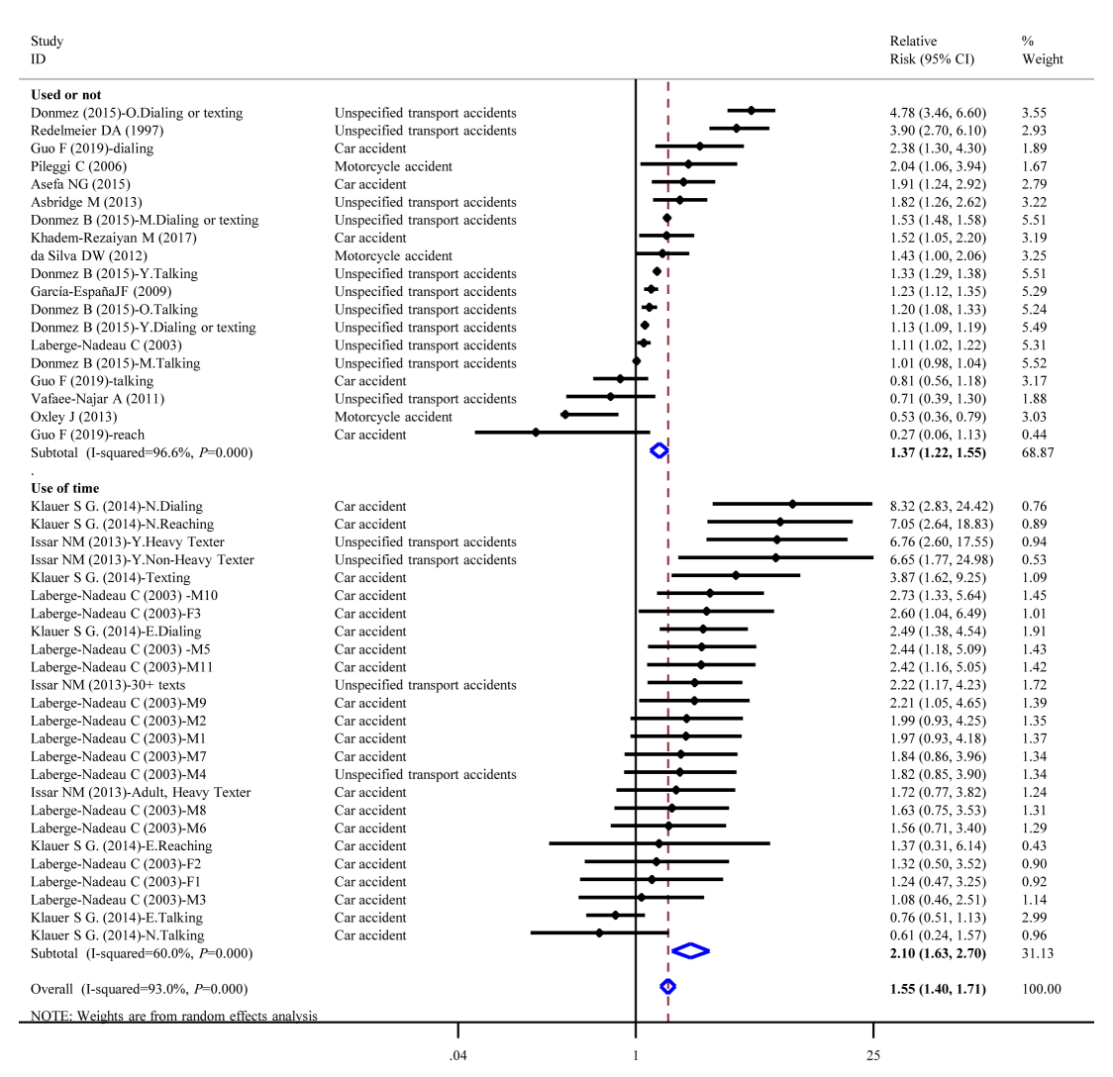
Forest plot of accident risk and mobile phone use.

Compared with nonmobile phone users, people who use mobile phones had a significantly higher risk for all accidents, with a pooled OR/RR of 1.55 (n=15,517,418, 95% CI 1.40 to 1.71; *I^2^*=93.7%). The risk for mobile phone users was 1.37 times (n=15,451,501‬, 95% CI 1.22 to 1.55; *I^2^*=96.6%) that for nonmobile phone users. The top 3 relative risks were 4.78 (95% CI 3.46 to 6.60) and 3.90 (95% CI 2.70 to 6.10), both for unspecified transport accidents, and 2.38 (95% CI 1.30 to 4.30) for car accidents. Those who used mobile phones long-term had a 2.10-fold (95% CI 1.63 to 2.70) higher risk of accidents than those who did not use mobile phones or who used them for short-term; the top 3 relative risks were 8.32 (95% CI 2.83 to 24.42), 7.05 (95% CI 2.64 to 18.83), and 6.76 (95% CI 2.60 to 17.55) for car accidents, car accidents, and unspecified transport accidents, respectively (Figure 2).

### Mobile Phone Use and Chronic Disorders

**Figure 3:**
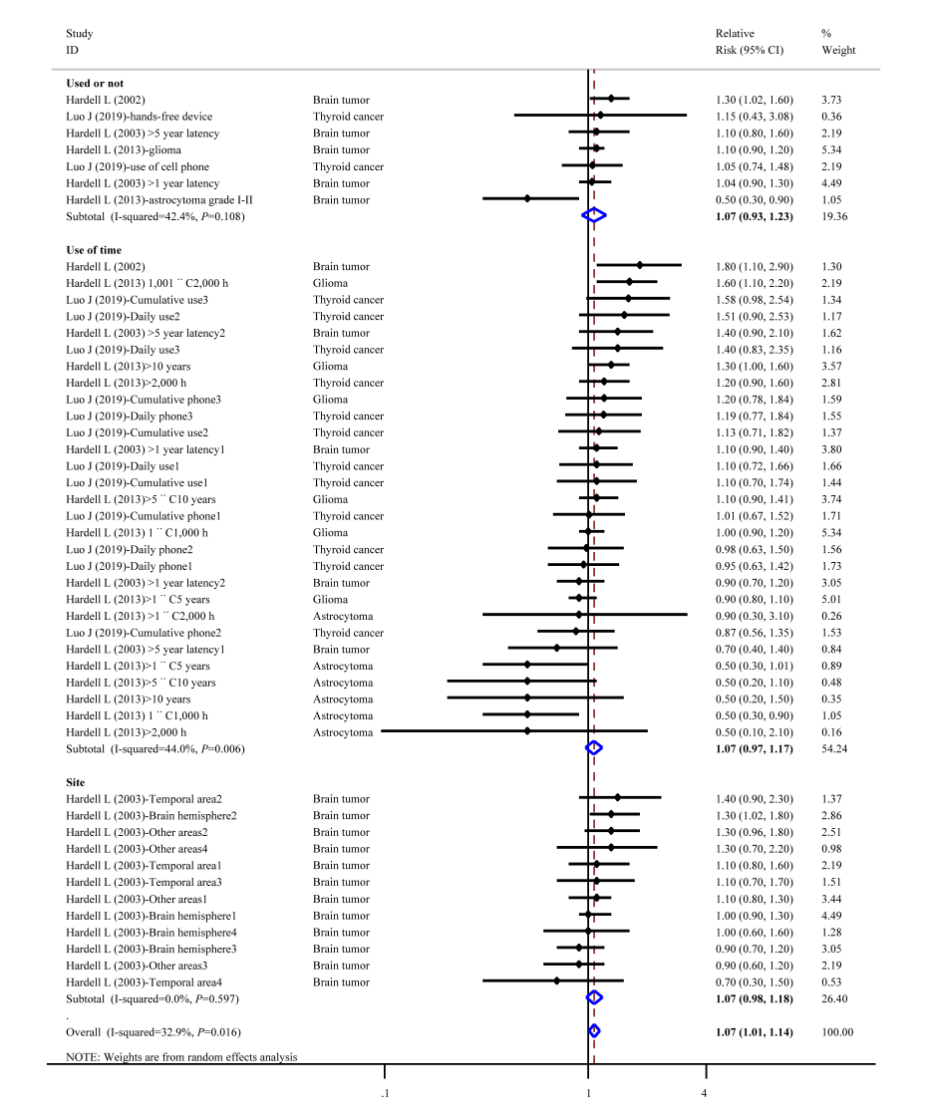
Forest plot of chronic disorder risk (neoplasms) and cell phone use.

The pooled risk of chronic disorders caused by mobile phone use was 1.07 times that of nonmobile phone use (95% CI 1.01 to 1.14; *I^2^*=32.9%). Compared with nonmobile phone users, mobile phone users had a 1.07-fold risk of neoplasms (95% CI 0.93 to 1.23, *I^2^*=42.4%). The top relative risks of neoplasms were for brain tumor (RR 1.30, 95% CI 1.02 to 1.60), followed by thyroid cancer (RR 1.15, 95% CI 0.93 to 1.23). For long-term mobile phone users there was a higher risk of neoplasms, with a pooled relative risk of 1.07 (95% CI 0.97 to 1.17). The top 3 relative risks for outcomes were brain tumor (RR 1.80, 95% CI 1.10 to 2.90), glioma (RR 1.60, 95% CI 1.10 to 2.20), and thyroid cancer (RR 1.58, 95% CI 0.98 to 2.54). Furthermore, the position when using mobile phone also increased the risk of specific cancers; mobile phone users had a relative risk of 1.40 (95% CI 0.98 to 1.18; *I^2^*=0.0%) for brain tumor compared with nonmobile phone users (Figure 3).

**Figure 4:**
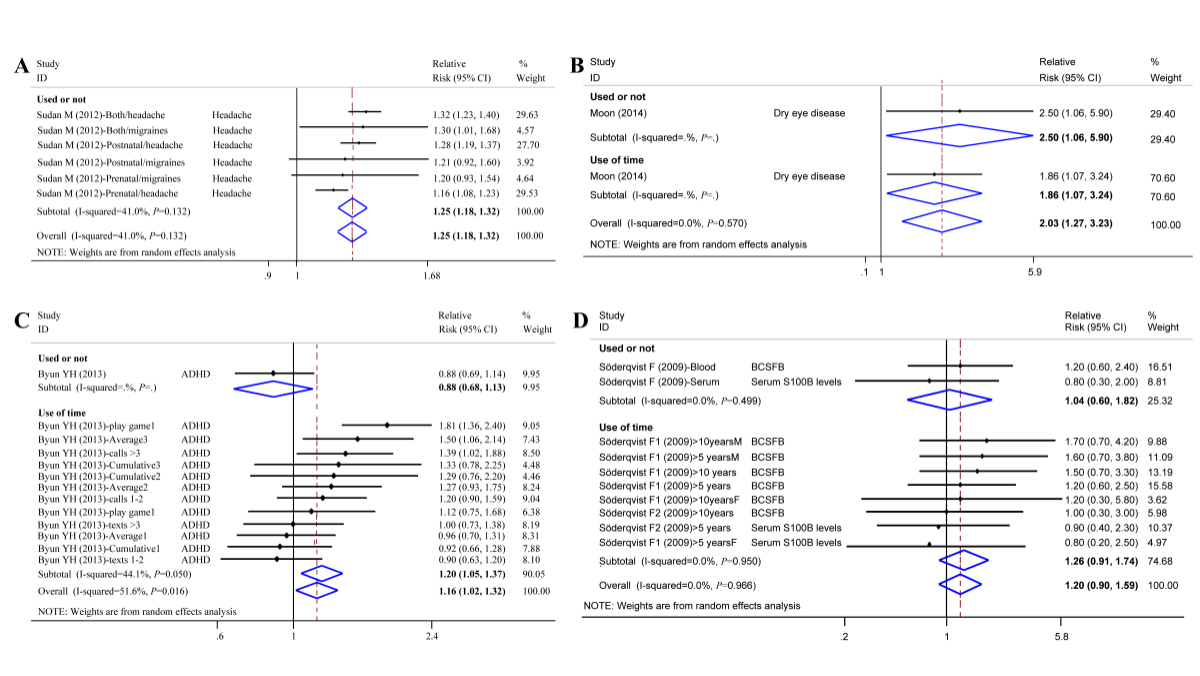
Forest plot of chronic disorder risk (nonneoplasm) and cell phone use. ADHD: attention deficit/hyperactivity disorder; BCSFB: blood-cerebrospinal fluid barrier.

Chronic nonneoplasm disorders caused by mobile phone use included mental disorders (ADHD, nomophobia), headaches, sleep disorders, injuries to the head (eye, ear, and oral), injuries to the wrist, male reproductive health issues, and other unspecific chronic disorders. Compared with nonmobile phone use, mobile phone use increased the risk of headaches (pooled risk 1.25, 95% CI 1.18 to 1.32, *I^2^*=41.0%; Figure 4A) and the risk of dry eye disease (RR 2.03, 95% CI 1.27 to 3.23, *I^2^*=0.0%; Figure 4B). Mobile phone users had a higher risk of ADHD than nonmobile phone users (RR 1.16, 95% CI 1.02 to 1.32, *I^2^*=51.6%; Figure 4C), and mobile phone use increased the risk of other unspecific chronic disorders, with a pooled risk of 1.20 (95% CI 0.90 to 1.59, *I^2^*=0.0%), including damage to the blood–cerebrospinal fluid barrier and elevated levels of serum S100B levels (Figure 4D).

**Figure 5:**
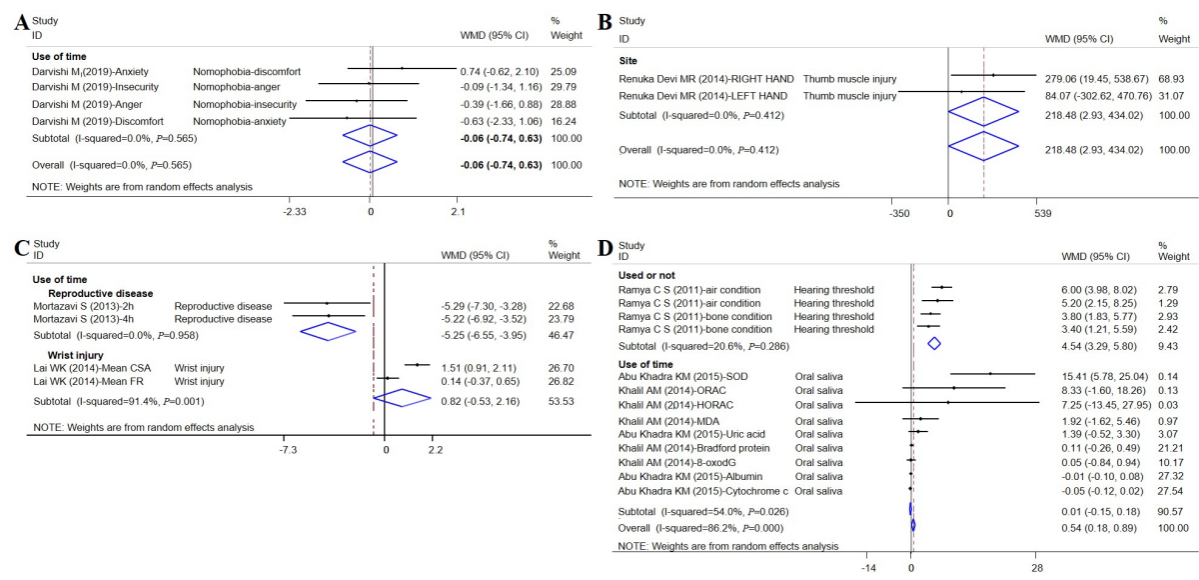
Forest plot of chronic disorder risk and cell phone use (continuous data). WMD: weight mean difference.

Compared to nonmobile phone users and short-term users, the risk for nomophobia among long-term users was –0.06 (95% CI –0.74 to 0.63; *I^2^*=0.0%; Figure 5A); the risk was not statistically significant. Mobile phone use increased the risk of thumb injury (weighted mean difference [WMD] 218.48, 95% CI 2.93 to 434.02; *I^2^*=0.0%; Figure 5B) and wrist extension (WMD 0.82, 95% CI –0.53 to 2.16; *I^2^*=91.4%; Figure 5C). The risk of damage to hearing was 4.54 times higher for mobile phone users than that of the nonmobile phone users (WMD 4.54, 95% CI 3.29 to 5.80, *I^2^*=20.6%; Figure 5D).

### Subgroup Analysis

Subgroup analysis showed a consistent increase in the overall risk of cancer in the population (Table 1). Participants from the United States (OR 1.35, 95% CI 1.18 to 1.55), Denmark (OR 1.25, 95% CI 1.18 to 1.32), and aged 18 to 35 years (OR 1.62, 95% CI 1.31 to 2.00) had higher risks of injury with mobile phone use. Similarly, the larger the sample size, the higher the risk of injury caused by the use of mobile phones. The risk of unspecified transport accidents significantly increased with mobile phone use as a result of accidents (OR 1.43, 95% CI 1.25 to 1.64). The higher risks of chronic disorders on the human body were injuries to the ear (OR 4.54, 95% CI 3.29 to 5.80), headaches (OR 1.25, 95% CI 1.18 to 1.32), and other unspecific chronic disorders (OR 0.51, 95% CI 0.04 to 0.99).

**Table 1:** Subgroup analyses of the risk of injuries by mobile phone use or nonuse.

Component			Studies, n (%)		Odds ratio (95% CI) (or random-effects weighted mean difference^a^)
**Country**					
	Iran	2 (2)		1.08 (0.51, 2.27)	
	Canada	3 (3)		1.95 (0.94, 4.07)	
	United States	5 (13)		1.35 (1.18, 1.55)	
	Denmark	1 (6)		1.25 (1.18, 1.32)	
	Sweden	4 (7)		1.06 (0.91, 1.24)	
**Sample size**					
	100-500	5 (8)		1.17 (0.79, 1.72)	
	500-1000	5 (6)		1.76 (1.14, 2.71)	
	>1000	10 (22)		1.21 (1.11, 1.32)	
**Age**					
	1-18 years	4 (9)		1.23 (1.15, 1.32)	
	18-35 years	4 (4)		1.62 (1.31, 2.00)	
	35-65 years	5 (7)		1.02 (0.87, 1.21)	
**Accidents**					
	Car accident	3 (5)		1.31 (0.81, 2.13)	
	Unspecified transport accidents	6 (11)		1.43 (1.25, 1.64)	
	Motorcycle accident	3 (3)		1.13 (0.51, 2.48)	
**Chronic disorders**					
	Mental disorders	2 (2)		1.37 (0.54, 3.51)	
	Headache	1 (6)		1.25 (1.18, 1.32)	
	Neoplasms	4 (7)		1.07 (0.93, 1.23)	
	Other unspecific chronic disorders	2 (2)		1.04 (0.60, 1.82)	
**Chronic disorders**					
	Other unspecific chronic disorders	2 (4)		0.51 (0.04, 0.99)^a^	
	Injuries to ear	1 (4)		4.54 (3.29, 5.80)^a^	
	DNA damage	1 (1)		0.13 (-0.15, 0.40)^a^	

^a^Outcome measures are continuous variables; therefore, random-effects weighted mean difference was used.

Among the participants with various mobile phone use duration, Canadians and Koreans had a higher risk of injury to the human body compared with that of other populations. In studies with a participant sample size that ranged from 100 to 500 and with participants aged 18 to 35 years, there was a higher risk of accidents and chronic disorders (Table 2). In general, mobile phone use increased the risk for injury to the human body. Similarly, unspecified transport accidents were the highest cause of human body injuries as a result of accidents (OR 3.23, 95% CI 1.65 to 6.30). Increasing mobile phone use was associated with the higher risks of DNA damage (OR 7.52, 95% CI 2.23 to 12.81), male reproductive health issues (OR –4.69, 95% CI –5.64 to –3.75), and mental disorders (OR 1.20, 95% CI 1.05 to 1.37).

**Table 2:** Subgroup analyses of the risk of injuries by the duration of mobile phone use.

Component			Studies (included entries), n (%)		Odds ratio (95% CI) (or random-effects weighted mean difference^a^)
**Country**					
	United States	3 (23)		1.20 (0.78, 1.84)	
	Canada	1 (14)		1.91 (1.54, 2.35)	
	Korea	1 (12)		1.20 (1.05, 1.37)	
	Sweden	4 (37)		1.06 (0.98, 1.15)	
**Sample size**					
	100-500	4 (19)		1.89 (1.32, 2.71)	
	500-1000	1 (12)		1.13 (0.99, 1.28)	
	>1000	5 (55)		1.16 (1.07, 1.25)	
**Age**					
	1-18 years	1 (12)		1.20 (1.05, 1.37)	
	35-65 years	6 (41)		1.16 (1.03, 1.30)	
**Accidents**					
	Car accident	2 (21)		1.95 (1.49, 2.55)	
	Unspecified transport accidents	1 (4)		3.23 (1.65, 6.30)	
**Chronic disorders**					
	Mental disorders	1 (12)		1.20 (1.05, 1.37)	
	Tumors	4 (41)		1.07 (1.00, 1.15)	
	Other unspecific chronic disorders	2 (8)		1.26 (0.91, 1.74)	
**Chronic disorders**					
	Nomophobia	1 (4)		–0.06 (–0.74, 0.63)^a^	
	Oral problem	2 (9)		0.01 (–0.15, 0.18)^a^	
	DNA damage	2 (4)		7.52 (2.23, 12.81)^a^	
	Male reproductive health issues	1 (4)		–4.69 (–5.64, –3.75)^a^	
	Injuries to wrist	1 (2)		0.82 (–0.53, 2.16)^a^	

^a^Outcome measures are continuous variables; therefore, random-effects weighted mean difference was used.

### Publication Bias

Research results that are statistically significant may be more likely to be reported and published than results that are insignificant and invalid. In our study, the funnel plot was generally symmetric, indicating the absence of publication bias (Figure S1 in Multimedia Appendix 1).

## Discussion

### Principal Findings

Our review included large participant-level cohort, cross-sectional, and case-control studies on the impact of mobile phone use on outcomes related to harm to the human body. The findings suggested that mobile phone use increased the risk of accidents and chronic disorders involving the human body. Mobile phone use increased the risk of accidents by 55%. Car accidents had the highest relative risk of traffic injuries for mobile phone users. Mobile phone use also increased the risk of chronic disorders, increasing the risk of neoplasms, ADHD, headaches, and eye injuries by 7%, 16%, 25%, and 103%, respectively.

Consistent with the findings of previous studies [66-69], mobile phone use while driving increased the risk of accidents, given that it may lead to decreased situational awareness and deteriorated driving performance. Phone use while driving has become a priority road safety issues, and although it is difficult to assess the absolute increased risk for collision due to distraction of drivers caused by using mobile phones, driving simulator [6] and real-world [67] naturalistic driving studies have shown that the risk for talking on the phone while driving is significantly higher than that for undistracted driving and is comparable to the risk of driving while drunk. Ludovic et al [70] found that mobile phone use while driving was a significant distraction—even when a user is not using a mobile phone, the vibration or beeping of the phone will attract the user's attention, thus becoming a cause of motor vehicle crashes. Drivers were more likely to miss traffic signals and were involved twice as often in car crashes when having a phone conversation while driving. In addition, visual manual tasks such as texting or typing were more likely to increase the risk of traffic accidents than other types of observable distractions [70,71]. Some interventional driving strategies and preventive measures have reduced the risk of traffic accidents among people, such as graduated driver licensing programs or advertising campaigns [72]. For example, United States, Great Britain, Canada, South Africa, and Australia have developed and use a graduated driver licensing program, which allows drivers to gain experience in low-risk driving conditions by adding an intermediate phase between the learning stage and the acquisition of the driving license [73]. Some studies [74,75] showed that the effectiveness of educational and preventive road safety programs is yet to be confirmed.

Although the risk of neoplasm from mobile phone use is still unclear, our meta-analysis suggests that improper use of mobile phones increases the risk of brain tumor, glioma, and thyroid cancer. Mobile phone radiation has been classified as possibly carcinogenic to humans [76]. There appears to be sufficient evidence that radiofrequency electromagnetic fields can cause nonthermal biological effects even when they do not cause tissue heating [77,78]. Previous evidence of damage from radiofrequency electromagnetic fields is the strongest for cancers caused by long-term exposure to mobile phones, especially brain tumor gliomas, glioblastomas, and acoustic neuromas [79,80]. In fact, the rates of brain tumors are increasing in Sweden, and the use of phones has been suggested to be the cause [81]. Little et al [82] found that ever having used mobile phones is not significantly associated with risk of glioma, but there could be increased risk for long-term users. Incorrect phone posture can increase the risk of wrist damage, chronic neck pain, and chronic shoulder pain, and the pain and fatigue worsen with longer mobile phone use [83-85]. When people use mobile phones, their body is relaxed, and their neck is prone to be bent. Hansraj et al [86] showed that there was a positive correlation between neck flexion and neck force, as well as head and neck posture in cervical spine stress and related neck pain. In addition, the long-term use of mobile phones may lead to ADHD in children and nomophobia. Studies have shown that adolescents with ADHD use electronic products significantly more often, and they usually have more sleep-wake problems [87]. Mobile phones are playing an increasingly important role in our lives. People have become dependent on mobile phones and suffer from *no mobile phone* *phobia* (ie, when not having a mobile phone, individuals feel discomfort, insecurity, anxiety, or anger), although the definition of *nomophobia* is not standardized, scholars have shown increasing interest and relevant scales have been designed and adjusted for different regions [88]. Finally, our meta-analysis demonstrated that mobile phone use can cause other chronic disorders, such as DNA damage (WMD 0.13, 95% CI –0.15 to 0.40). Several studies [89,90] have shown that radiofrequency radiation exposure can lead to oxidative stress in various tissues. Oxidative stress is known to play a central role in the development of cancer and aging, and it serves as a signaling agent in the inflammatory response. Recent studies [15,91] reported that the radiofrequency radiation emitted from mobile phones causes oxidative stress. Oxidative stress related to radiofrequency radiation leads to lipid, protein, and DNA damage in various tissues [15]. Our findings suggest that although the current allowable mobile phone radiation level is very low, it may be sufficient to induce biological effects. Some studies [82,92,93] have reported that existing data are not sufficient to support the assumption that tumors are caused by mobile phone usage. Thus, determination of whether these effects might cause any significant health effects requires further investigation, especially with respect to neoplasms.

### Limitations

The inclusion criteria for our study were rigorous, and thus, some reports were excluded. For example, the incidences of taking selfies and sharing them on social media as well as selfie-related behaviors are increasing, particularly among young people, which possibly leads to selfie-related trauma [94,95]. Other studies [96,97,98] have reported physical harm caused by mobile phones, such as ear trauma, thigh injuries, electrical burns, and injuries caused by phone explosions. Furthermore, it has been suggested that electromagnetic fields generated by mobile phone may have long-term harmful effects, including an increase in infertility, Alzheimer disease, and other neurodegenerative diseases [99].

Our study also has some limitations. First, “damage” and “injury” were used as search queries in our study to retrieve papers on the health effects of mobile phones, other adverse outcomes caused by phone use may have been missed. Second, only 10 of the 41 studies were longitudinal studies. Additional longitudinal studies could confirm the causal relationship between mobile phone use and human health. Third, the different environments and behaviors of using mobile phones might lead to different risks of injury. We did not consider different patterns or reasons for using mobile phones in different regions and by different people, and we did not further analyze specific types and purposes of using mobile phones, such as texting or making phone calls. Finally, there was heterogeneity in our study (*I*^2^>75%); therefore, we performed subgroup analyses to explore the source of heterogeneity.

### Conclusions

There is growing evidence that mobile phone use affects the human body. Our study suggests that the use of mobile phones causes not only accidents but also chronic disorders to the human body. Although some findings are still controversial, the harm that mobile phones cause to the human body cannot be underestimated, and more research is needed to explore the direct evidence of damage to the human body.

## Appendix

Multimedia Appendix 1 Literature search strategy, basic characteristics of studies, quality assessment, and publication bias test.

## Abbreviations

ADHD: attention-deficit/hyperactivity disorder
fPSA: free prostate specific antigen
OR: odds ratio
RR: relative risk
tPSA: total prostate specific antigen
WMD: weighted mean difference
