# Supplementary material for: Risk of Accidents or Chronic Disorders From Improper Use of Mobile Phones: A Systematic Review and Meta-analysis
Source: J Med Internet Res. 2022 Jan 20;24(1):e21313. doi: 10.2196/21313 (PMC8814932; doi:10.2196/21313)
Supplement: Multimedia Appendix 1 [file jmir_v24i1e21313_app1.pdf]

Supplementary Appendix

This appendix has been provided by the authors to give readers additional information about their work.

Supplement to: Xinxi Cao, Yangyang Cheng, Chenjie Xu, et al.
Risk of Accidents or Chronic Disorders From Improper Use of Mobile Phones: A Systematic Review and Meta-analysis

Table S1 Literature Search Strategy

S1.1 Literature Search Strategy for Database PubMed (Through Apr 4, 2019)

	**Search Terms**	**Results**
#1	Search ("Cell Phone"[Mesh]) OR "Cell Phone Use"[Mesh]	9,384
#2	Search (((((((((((((((((((((((((((((((cell phone[Title/Abstract]) OR Phone, Cell[Title/Abstract]) OR Phones, Cell[Title/Abstract]) OR Cellular Phone[Title/Abstract]) OR Cellular Phones[Title/Abstract]) OR Phone, Cellular[Title/Abstract]) OR Phones, Cellular[Title/Abstract]) OR Telephone, Cellular[Title/Abstract]) OR Cellular Telephone[Title/Abstract]) OR Cellular Telephones[Title/Abstract]) OR Telephones, Cellular[Title/Abstract]) OR Cell Phones[Title/Abstract]) OR Portable Cellular Phone[Title/Abstract]) OR Cellular Phone, Portable[Title/Abstract]) OR Cellular Phones, Portable[Title/Abstract]) OR Portable Cellular Phones[Title/Abstract]) OR Transportable Cellular Phone[Title/Abstract]) OR Cellular Phone, Transportable[Title/Abstract]) OR Cellular Phones, Transportable[Title/Abstract]) OR Transportable Cellular Phones[Title/Abstract]) OR Mobile Phone[Title/Abstract]) OR Mobile Phones[Title/Abstract]) OR Phone, Mobile[Title/Abstract]) OR Phones, Mobile[Title/Abstract]) OR Mobile Telephone[Title/Abstract]) OR Mobile Telephones[Title/Abstract]) OR Telephone, Mobile[Title/Abstract]) OR Telephones, Mobile[Title/Abstract]) OR Car Phone[Title/Abstract]) OR Car Phones[Title/Abstract]) OR Phone, Car[Title/Abstract]) OR Phones, Car[Title/Abstract]	13,041
#3	Search (((((((((Cell phone use[Title/Abstract]) OR Cell Phone Uses[Title/Abstract]) OR Phone Use, Cell[Title/Abstract]) OR Use, Cell Phone[Title/Abstract]) OR Uses, Cell Phone[Title/Abstract]) OR Mobile Phone Use[Title/Abstract]) OR Mobile Phone Uses[Title/Abstract]) OR Phone Use, Mobile[Title/Abstract]) OR Use, Mobile Phone[Title/Abstract]) OR Uses, Mobile Phone[Title/Abstract]	10,681
#4	Search (#1 OR #2 OR #3)	16,595
#5	Search ((((((((((Hurt*[Title/Abstract]) OR Injur*[Title/Abstract]) OR Harm*[Title/Abstract]) OR Disadvantage*[Title/Abstract]) OR Endanger*[Title/Abstract]) OR Jeopard*[Title/Abstract]) OR Damage*[Title/Abstract]) OR Threat*[Title/Abstract]) OR Menace[Title/Abstract]) OR Imperil*[Title/Abstract]	1,482,550
#6	Search (#4 AND #5)	1,254

S1.2 Literature Search Strategy for Database Embase (Through Apr 4, 2019)

	Search Terms	Results
#1	'cell phone':ab,ti OR 'phone, cell':ab,ti OR 'phones, cell':ab,ti OR 'cellular phone':ab,ti OR 'cellular phones':ab,ti OR 'phone, cellular':ab,ti OR 'phones, cellular':ab,ti OR 'telephone, cellular':ab,ti OR 'cellular telephones':ab,ti OR 'telephones, cellular':ab,ti OR 'cell phones':ab,ti OR 'portable cellular phone':ab,ti OR 'cellular phone, portable':ab,ti OR 'cellular phones, portable':ab,ti OR 'portable cellular phones':ab,ti OR 'transportable cellular phone':ab,ti OR 'cellular phone, transportable':ab,ti OR 'cellular phones, transportable':ab,ti OR 'transportable cellular phones':ab,ti OR 'mobile phone':ab,ti OR 'mobile phones':ab,ti OR 'phone, mobile':ab,ti OR 'phones, mobile':ab,ti OR 'mobile telephone':ab,ti OR 'mobile telephones':ab,ti OR 'telephone, mobile':ab,ti OR 'telephones, mobile':ab,ti OR 'car phone':ab,ti OR 'car phones':ab,ti OR 'phone, car':ab,ti OR 'phones, car':ab,ti OR 'cellular telephone':ab,ti	12.640
#2	'cell phone use':ab,ti OR 'cell phone uses':ab,ti OR 'phone use, cell':ab,ti OR 'use, cell phone':ab,ti OR 'uses, cell phone':ab,ti OR 'mobile phone use':ab,ti OR 'mobile phone uses':ab,ti OR 'phone use, mobile':ab,ti OR 'use, mobile phone':ab,ti OR 'uses, mobile phone':ab,ti	1,174
#3	#1 OR #2	12,640
#4	hurt*:ab,ti OR injur*:ab,ti OR harm*:ab,ti OR disadvantage*:ab,ti OR endanger*:ab,ti OR jeopard*:ab,ti OR damage*:ab,ti OR threat*:ab,ti OR menace:ab,ti OR imperil*:ab,ti	1,896,075
#5	#3 AND #4	1,140

S1.3 Literature Search Strategy for Database Web of Science (Through Apr 4, 2019)

	Search Terms	Results
#1	TS=("cell phone" OR "Phone, Cell" OR "Phones, Cell" OR "Cellular Phone" OR "Cellular Phones" OR "Phone, Cellular" OR "Phones, Cellular" OR "Telephone, Cellular" OR "Cellular Telephone" OR "Cellular Telephones" OR "Telephones, Cellular" OR "Cell Phones" OR "Portable Cellular Phone" OR "Cellular Phone, Portable" OR "Cellular Phones, Portable" OR "Portable Cellular Phones" OR "Transportable Cellular Phone" OR "Cellular Phone, Transportable" OR "Cellular Phones, Transportable" OR "Transportable Cellular Phones" OR "Mobile Phone" OR "Mobile Phones" OR "Phone, Mobile" OR "Phones, Mobile" OR "Mobile Telephone" OR "Mobile Telephones" OR "Telephone, Mobile" OR "Telephones, Mobile" OR "Car Phone" OR "Car Phones" OR "Phone, Car" OR "Phones, Car")	17,303
#2	TS=(Cell phone use OR Cell Phone Uses OR Phone Use, Cell OR Use, Cell Phone OR Uses, Cell Phone OR Mobile Phone Use OR Mobile Phone Uses OR Phone Use, Mobile OR Use, Mobile Phone OR Uses, Mobile Phone)	12,134
#3	#1 OR #2	18,644
#4	TS=(Hurt* OR Injur* OR Harm* OR Disadvantage* OR Endanger* OR Jeopard* OR Damage* OR Threat* OR Menace OR Imperil*)	1,778,599
#5	#3 AND #4	1,494

S1.4 Literature Search Strategy for Database Cochrane Library (Through Apr 4, 2019)

	Search Terms	Results
#1	cell phone OR Phone, Cell OR Phones, Cell OR Cellular Phone OR Cellular Phones OR Phone, Cellular OR Phones, Cellular OR Telephone, Cellular OR Cellular Telephone OR Cellular Telephones OR Telephones, Cellular OR Cell Phones OR Portable Cellular Phone OR Cellular Phone, Portable OR Cellular Phones, Portable OR Portable Cellular Phones OR Transportable Cellular Phone OR Cellular Phone, Transportable OR Cellular Phones, Transportable OR Transportable Cellular Phones OR Mobile Phone OR Mobile Phones OR Phone, Mobile OR Phones, Mobile OR Mobile Telephone OR Mobile Telephones OR Telephone, Mobile OR Telephones, Mobile OR Car Phone OR Car Phones OR Phone, Car OR Phones, Car	3,786
#2	Cell phone use OR Cell Phone Uses OR Phone Use, Cell OR Use, Cell Phone OR Uses, Cell Phone OR Mobile Phone Use OR Mobile Phone Uses OR Phone Use, Mobile OR Use, Mobile Phone OR Uses, Mobile Phone	2,475
#3	#1 OR #2	3,786
#4	Hurt* OR Injur* OR Harm* OR Disadvantage* OR Endanger* OR Jeopard* OR Damage* OR Threat* OR Menace OR Imperil*	90,667
#5	#3 AND #4	337

Table S2 Basic characteristics of 41 studies included.

First author (year)	Study area, country	Sample size	Sample age (yrs, range and/or mean±SD)	Statistical model	Measures of cell phone usage	Other factors adjusted for in the model	Measures of outcome-related behavior	Outcomes
**Transport injury**								
**Issar (2013)[19]**	Nashville, US	177	Group A:38.0<br>Group B:44.4	Logistic models	Cell phone usage	NA	Motor vehicle collisions, and automobile involvement in trauma	Motor vehicle collision frequency
**Truong (2019)[20]**	Vietnam	665	M=21.9 in 2016	Binary logistic regression	Using a mobile phone (for calling, texting, and searching for information)	NA	Whether involved in a crash/fall while riding a motorcycle	Motorcycle and crashesy
**García-España (2009)[21]**	US	2,167	Shared Access:16.7±0.06<br>Primary Access:17.0±0.06	Multivariable logistic regression model	Use cellular telephones while driving	Vehicle type, age, grade, gender, race/ethnicity, urbanicity, school grades, socioeconomic level, working at a job, and reported hours per week of driving	Crash Involvement	Driving Behaviors and Crashes
**Pileggi (2006)[22]**	Italy	894	M=17.2 in 2004	Multiple logistic regression models	Used cell phones while motorcycling	NA	Behavior while driving a motorcycle; traffic-related accidents	Risky behaviors among motorcycling
**Labergenadeau (2003)[23]**	Canada	36,078	16-64 in 1996-1999	Multiple logistic regression models	Whether use cell phone when driving	Age	At least one accident in a given year	Road crashes
**Donmez (2015)[34]**	US	15,406,515	NA	Logit model	Talking on a cell phone, dialing/texting on a cell phone while driving	Environmental conditions, crash profile, vehicle model year, and driver information	Driving injury severity	Driver injury

**Oxley (2013)[35]**	Malaysia	1,750	>18	Hierarchical logistic regression model	Checking sms while riding	NA	Crash involvement	Commuter motorcycle crashes
**Vafaeenajar (2011)[36]**	Iran	312	37.4±10.3 in 2007	Logistic regression model	Whether or not use hand phone while driving	NA	Accidents resulted in casualties<br>Accidents without casualties	Accidents injuries
**Redelmeier (1997)[38]**	Toronto, Canada	699	NA	Conditional logistic-regression	Texting or using Internet<br>Dialing Talking<br>Reaching for phone	NA	Motor Vehicle Crash or Near-Crash	Road Crashes
**Guo (2019)[39]**	US	102	NA	Semiparametric Bayesian model with logit link	Type of cellular telephone when driving	Age, sex, visual acuity, training, personality, driving record	Motor Vehicle Collision	Motor vehicle collisions
**Klauer (2014)[53]**	US	151	36.2±14.4	Logistic-regression analysis	Driver behavior, such as distraction	NA	Traffic crashes	Driving risk
**Khadem-Rezaiyan (2017)[43]**	Mashhad, Iran	431	30±11.3 in 2014	Logistic regression	Whether or not using a cell phone	NA	History of previous car accidents	Driving risk
**Asbridge (2013)[45]**	British Columbia, Canada	1,091	Above 16 in 2005–2008	Logit regression	Whether or not using a cell phone at the time of the crash	Age, licence status	Drive crashes	Traffic crash
**da Silva (2012)[46]**	Brazil	750	29.5±8.1 in 2005	Multivariate analysis	Use of cell phone while driving	NA	Road accidents	Road Traffic Crash
**Asefa (2015)[47]**	Vietnam	712	26 (19-60) in 2014	Multivariate logistic regression	Method of receiving mobile calls when driving	NA	Involved in a road traffic crash	Road Traffic Crash
**Chronic injury**								

**Zhang (2016)**[14]	Chongqing, China	2,028	20.4±1.2 in 2013	Multilevel linear regression	The duration of cell phone use	Including age, duration of abstinence, BMI, smoking and drinking status, and cola, coffee, and fried food consumption	The position in which they carry the cell Phone<br>Talking on the cell phone<br>Internet use via cellular network	Male reproductive health
**Ryan (2005)**[15]	Massachusetts, US	153	36.0 in 2008	Linear mixed-effects models	Mobile phone use duration (use or not) and location	Age, race, BMI, and abstinence time	Semen was collected on site at MGH	Male reproductive health
**Byun (2013)**[16]	Korea	2,422	8.94±0.74 in 2008	Logistic regression	The ownership of a mobile phone by children<br>Mobile phone accessibility	Age, gender, number of siblings, residential area, household income, maternal smoking during pregnancy, child's history of neuropsychiatric illness, parental marital status, parental history of neuropsychiatric disease, blood lead levels	Measured Blood Lead<br>ADHD rating scale	Attention Deficit Hyperactivity Symptoms
**Sudan (2012)**[17]	Denmark	52,680	Children in 7 years	Logistic regression models	Whether or not the mother used a cell phone while she was pregnantWhether or not children use a cell phone	Age, mother's history of migraines, mother's feelings of worry, burden, and stress during pregnancy, social occupational status, child's exposure to tobacco smoke, and child's sex	Measured migraines	Headaches
**Simşek (2003)**[18]	NA	20	33.9 ± 11.4 in 2001	Wilcoxon paired signed ranks test	Carry cellular telephones constantly in the on position for 30 days	NA	Free and total PSA levels	Serum PSA levels
**Souza (2014)**[24]	NA	45	1: 32.73±9.03<br>2: 31.87±9.78<br>3: 32.53±9.61	Conditional Test	Mean length of time spent on conversations using mobile phones per week	NA	Oral mucosa cells	Oral epithelium

**Khalil (2014)**[25]	NA	12	M=22	ANOVA	Before they started cell phone call, after 15 min and 30 min directly at the end of the call	NA	Determination of 8-OxodG<br>Determination of MDA<br>The salivary oxidative stress indices HORAC and ORAC	Oral saliva
**Luo (2018)**[26]	Connecticut, US	960	21-84 in 2010 and 2011	Multivariate unconditional logistic regression	The frequency, duration, and protective behaviors of cell phone use	Age, sex, education, family history of thyroid cancer, alcohol consumption, body mass index, previous benign thyroid diseases, occupational radiation exposure, and radiation treatment	Histologic subtype and tumor size, thyroid cancer	Thyroid cancer
**Hardell (2002)**[27]	Sweden	2,606	20-80, M=54	Conditional logistic regression analysis	Type of phone, years of use and brand name	NA	Brain tumour	Brain tumour
**Khadra (2015)**[49]	NA	12	M=22	ANOVA	Before the start of call as well as 15 min and 30 min immediately after calls	NA	Biochemical status in the saliva before/after using mobile phone	Oral saliva
**Hardell (2013)**[50]	Sweden	2,466	20-80, M=55	Cox proportional model	Type, time period, average number of minutes per day over the years, ear mostly used during calls (not for deceased subjects), use of hands-free devices and use of	Age, gender, year of diagnosis, socioeconomic code and study	The survival of patients with a malignant brain tumour in relation to the use of wireless phones	Glioma

					external antenna in a car			
**Tiwari (2008)**[28]	NA	6	20-24	Student’s t test	Radio frequency (RF) signal from Code Division Multiple Access (CDMA) mobile phones	NA	Assess the DNA damage	DNA Integrity
**Gadhia (2003)**[29]	NA	48	Group 1: M=32<br>Group 2: M=30	Student’s t test	Using digital mobile phones over the last 2 years	NA	Chromosomal aberrations	Chromosomal Damage
**Çam (2012)**[30]	NA	8	30-47	Paired t test	Radio frequency radiation(RFR)	NA	Measure DNA breaks	Single-strand DNA breaks
**Lai (2014)**[31]	Hong Kong, China	31	21.7±1.5	ANCOVA	Simulate text messaging<br>Rapid keying task using the same mobile phone	NA	Wrist extension	Wrist extension
**Renuka Devi (2014)**[32]	NA	50	NA	ANOVA	Text messaging	NA	Repetitive stress injury (RSI)	Thumb Muscle
**Mortazavi (2013)**[37]	NA	30	NA	T-test and analysis of variance	Radio frequency (RF) radiations	NA	Total motility score in sperms	Male reproductive health
**Zeni (2005)**[33]	NA	10	32.4±2.7	Student’s t test	Exposure to 900 MHz RF Field	NA	Chromosome aberrations	Genotoxic Effects
**Ramya (2011)**[54]	NA	50	15-40	Paired t test	Time of use mobile phones	NA	Hearing threshold	Hearing

**Hardell (2003)[40]**	Sweden	3,234	median age: 54 years	Logistic regression analysis	Type of phone<br>Years of cell phone use<br>Mean number of daily calls and minutes	Age, sex and SEI	Report histopathology diagnosis of brain tumour	Hearing
**Söderqvist[1] (2009)[41]**	Örebro, Sweden	313	18-65 in 2007	Unconditional logistic regression	Tested long-term and/or short-term use of wireless telephones	Age adjusted for in all analyses<br>Sex adjusted in analyses of total samples	Blood samples were analyzed for serum transthyretin concentrations	Blood-cerebrospinal fluid barrier
**Söderqvist[2] (2009)[42]**	Örebro, Sweden	314	41.7(18–65) in 2007	Unconditional logistic regression	Use of wireless phones	Gender and time of blood sampling	Serum S100B levels	Serum S100B levels
**Darvishi (2019)[44]**	NA	100	23.5±4.9 in 2016-2017	ANOVA	Average duration of mobile use	NA	Measured discomfort, anger, anxiety, and insecurity	Nomophobia
**Fuller (2017)[48]**	Pennsylvania, US	207	8-17 in 2016	Logistic regression	Access to cell phone at bedtime	NA	Measured BMI<br>Participants to be tired	Sleep Problems<br>Overweight
**Mortazavi Joowon (2016)[51]**	Korea	715	15 in 2013	Logistic regression models	The average number of hours	Age, sex, daily sleep duration, use of contact lenses, computer use and city	Symptoms in ocular health	Ocular Health
**Moon (2014)[52]**	Korea	288	Dry: 11.00±0.61<br>Normal: 10.87±0.66	Linear regression	Smartphones and use time per day	NA	Ocular symptoms for dry eye	Dry eye disease

Table S3 Quality assessment

S3.1. Quality assessment of included case-control studies in the Meta-analysis using the Newcastle-Ottawa Scale (NOS)#

Source	Case Definition	Representativeness of the Cases	Selection of Controls	Definition of Controls	Comparability of Cases and Controls	Ascertainment of Exposure	Same Method of Ascertainment for Cases and Controls	Nonresponse Rate	Total NOS Score
Souza (2014)	*	*	*	*	*	*	*	*	8
Khalil (2014)	*	*	*	*		*	*	*	7
Luo (2018)	*	*		*	**	*	*	*	8
Hardell (2002)	*	*		*	*	*	*	*	7
Abu Khadra (2014)	*	*	*	*	*	*	*	*	8
Hardell (2013)	*	*		*	*	*	*	*	7
Tiwari (2008)	*	*	*	*	*	*	*	*	8
Zeni (2005)	*		*	*		*	*	*	6
Gadhia (2003)	*	*	*	*	*	*	*	*	8
Çam (2012)	*	*	*	*	*	*	*	*	8
Lai (2014)	*	*	*	*	*	*	*	*	8
Renuka Devi (2014)	*	*	*	*	*	*	*	*	8
Donmez (2015)		*	*	*		*	*	*	6
Oxley (2013)	*	*	*	*		*	*	*	7
Vafaeenajar (2011)	*	*	*		*	*	*	*	7
Mortazavi (2013)	*	*	*	*	*	*	*	*	9
Redelmeier (1997)	*	*	*	*	**	*	*	*	9
Guo (2019)	*	*	*	*	*	*	*	*	8
Siebe (2014)	*	*	*	*		*	*	*	7

**Ramya (2011)**	*	*	*	*		*	*	*	**7**
**Hardell (2003)**	*	*	*	*	**	*	*	*	**9**
**Asbridge (2013)**	*	*	*	*	**	*	*	*	**9**

Article quality was assessed as follows: low quality = 0–5; moderate quality = 6–7; high quality = 8–9.

S3.2. Quality assessment of included cohort studies in the Meta-analysis using the Newcastle-Ottawa Scale (NOS)#

**Source**	**Representativeness of the exposed cohort**	**Selection of the non exposed cohort**	**Ascertainment of exposure**	**Demonstration that outcome of interest was not present at start of study**	**Comparability of cohorts**	**Assessment of outcome**	**follow-up long enough**	**Adequacy of follow up of cohorts**	**Total NOS Score**
**Zhang (2016)**	*	*	*		*	*	*	*	**7**
**Ryan (2017)**	*	*	*	*	**	*	*	*	**9**
**Byun (2013)**	*	*	*		*	*	*	*	**7**
**Sudan (2011)**	*	*	*	*	*	*	*		**7**
**Simşek (2003)**	*	*	*	*	*	*		*	**7**
**Issar (2013)**	*		*	*	*	*		*	**6**
**Truong (2019)**	*	*	*	*	*	*		*	**7**
**García-España (2009)**	*	*	*	*	*	*		*	**7**
**Pileggi (2006)**	*	*	*	*	*	*		*	**7**
**Labergenadeau (2003)**	*	*	*	*	*	*		*	**7**

Article quality was assessed as follows: low quality = 0–5; moderate quality = 6–7; high quality = 8–9.

S3.3. Quality assessment of included cross-sectional studies in the Meta-analysis Using Agency for Healthcare Research and Quality (AHRQ) Standards#

Source	Söderqvist F[1] (2009)	Söderqvist F[2] (2009)	Rezaiyan (2017)	Darvishi (2019)	da Silva (2012)	Asefa (2015)	Fuller (2017)	Mortazavi Joowon (2016)	Moon(2014)
**Define the source of information**	*	*	*	*	*	*	*	*	*
**Inclusion and exclusion criteria**	*	*	*	*			*	*	*
**Time period used for identifying patients**	*	*			*	*	*	*	*
**Subjects were consecutive**	*	*	*	*	*	*	*	*	*
**If subjective components of study were masked**									
**Assessments for quality assurance purposes**	*	*				*	*		*
**Explain any patient exclusions from analysis**	*	*	*	*	*		*	*	*
**Describe how confounding was assessed and/or controlled**	*	*	*		*			*	
**Explain how missing data were handled in the analysis**									
**Summarize patient response rates and completeness**	*	*	*	*	*	*	*	*	*
**Clarify follow-up was obtained**									
**Total Scores**	**8**	**8**	**6**	**5**	**6**	**5**	**7**	**7**	**7**

Article quality was assessed as follows: low quality = 0–3; moderate quality = 4–7; high quality = 8–11.

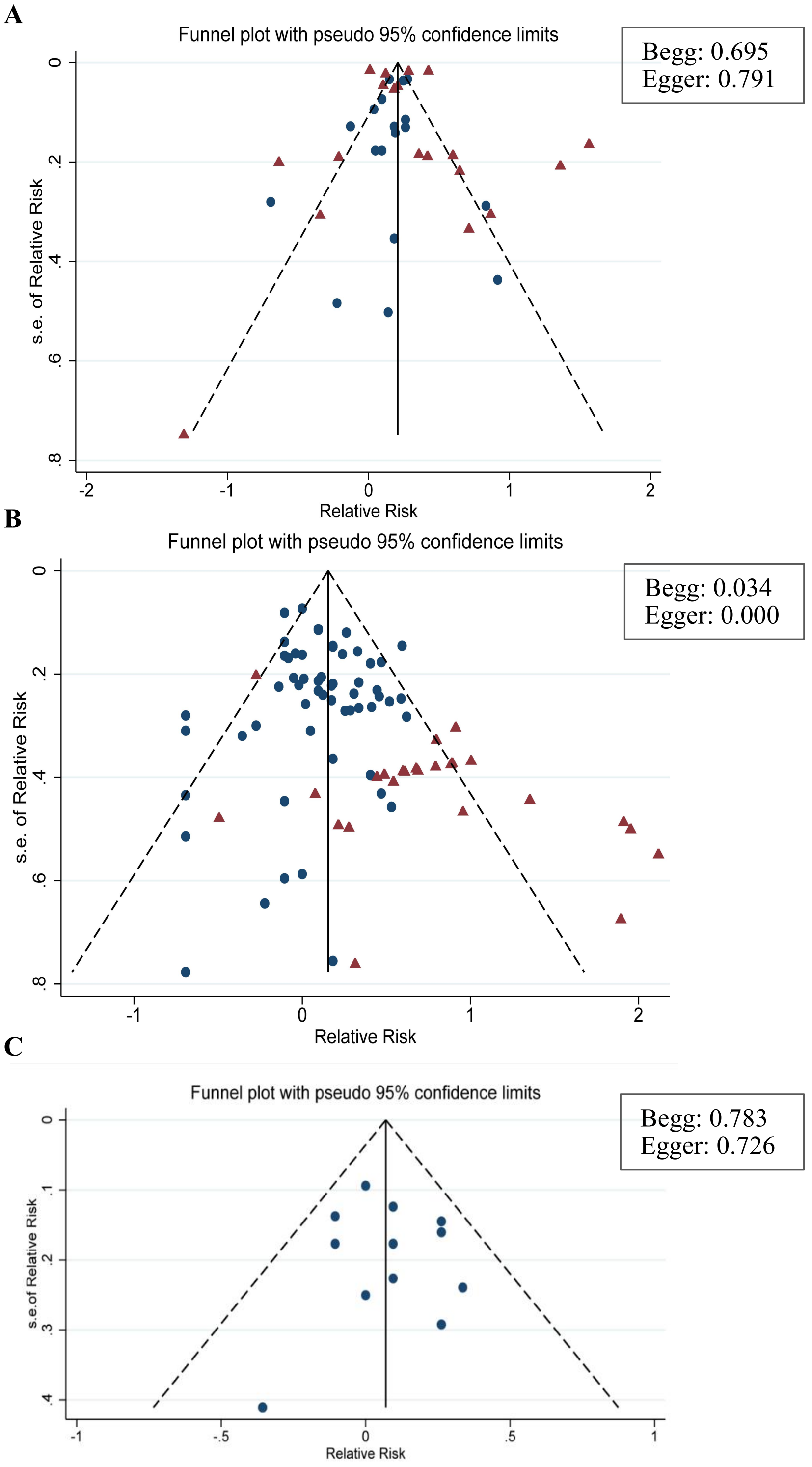

Figure S1 Funnel plot with pseudo 95% confidence limits (Begg's test and Egger's test).
(Panel A shows cell phone use or not and transport and chronic injury; Panel B shows cell phone use duration and transport and chronic injury; Panel C shows cell phone use site and chronic injury.)
